# Supplementary material for: Mechanisms governing the pioneering and redistribution capabilities of the non-classical pioneer PU.1
Source: Nat Commun. 2020 Jan 21;11:402. doi: 10.1038/s41467-019-13960-2 (PMC6972792; doi:10.1038/s41467-019-13960-2)
Supplement: Supplementary file 7 — Source data [file 41467_2019_13960_MOESM7_ESM.zip › Source_Data/FigureS2/FigureS2B_MotifScanOutput/KmeansCluster10/homerResults/motif24.similar.html]

motif24

## Information for motif24

T
G
C
A
A
C
G
T
C
T
A
G
C
G
T
A
A
C
T
G
C
A
G
T
G
A
T
C
G
C
T
A
A
C
G
T
A
G
T
C
G
C
A
T
  
Reverse Opposite:  

C
G
T
A
T
C
A
G
T
G
C
A
C
G
A
T
C
T
A
G
G
T
C
A
T
G
A
C
C
G
A
T
A
G
T
C
C
G
T
A
A
C
G
T
  

|  |  |
| --- | --- |
| p-value: | 1e-27 |
| log p-value: | -6.256e+01 |
| Information Content per bp: | 1.628 |
| Number of Target Sequences with motif | 288.0 |
| Percentage of Target Sequences with motif | 7.76% |
| Number of Background Sequences with motif | 1633.4 |
| Percentage of Background Sequences with motif | 3.70% |
| Average Position of motif in Targets | 103.8 +/- 55.0bp |
| Average Position of motif in Background | 99.3 +/- 64.0bp |
| Strand Bias (log2 ratio + to - strand density) | -0.2 |
| Multiplicity (# of sites on avg that occur together) | 1.04 |
| Motif File: | file (matrix) reverse opposite |

### Similar de novo motifs found

|  |  |  |  |  |  |  |  |
| --- | --- | --- | --- | --- | --- | --- | --- |
| Rank | Match Score | Redundant Motif | P-value | log P-value | % of Targets | % of Background | Motif file |
| 1 | 0.917 | A T C G T G C A A G C T C A T G G C T A A T G C C G A T A G T C C G T A A C G T | 1e-25 | -59.213777 | 10.08% | 5.46% | motif file (matrix) |
| 2 | 0.916 | A T C G T G C A C G A T A C T G C G T A T A G C C G A T A G T C C G T A A C T G G A T C G T C A | 1e-24 | -57.421277 | 6.28% | 2.82% | motif file (matrix) |
| 3 | 0.912 | A C G T C A T G G C T A T A C G A C G T A T G C C G T A A G C T T A G C | 1e-23 | -54.276341 | 8.43% | 4.41% | motif file (matrix) |
| 4 | 0.929 | A C G T A C T G C G T A T A C G A C G T A G T C C T G A A G C T | 1e-23 | -53.038585 | 10.43% | 5.95% | motif file (matrix) |
| 5 | 0.906 | A C G T A C T G C G T A C T A G A C G T A G T C C G T A | 1e-18 | -43.168678 | 6.73% | 3.53% | motif file (matrix) |
